# Pulmonary Hypertension and the Risk of 30-Day Postoperative Pulmonary Complications after Gastrointestinal Surgical or Endoscopic Procedures: A Retrospective Propensity Score-Weighted Cohort Analysis

**DOI:** 10.3390/jcm13071996

**Published:** 2024-03-29

**Authors:** Yoshio Tatsuoka, Zyad J. Carr, Sachidhanand Jayakumar, Hung-Mo Lin, Zili He, Adham Farroukh, Paul Heerdt

**Affiliations:** 1 Department of Anesthesiology, Yale University School of Medicine, New Haven, CT 06510, USA; yoshio.tatsuoka@yale.edu (Y.T.);; 2 Department of Anesthesiology, Yale New Haven Hospital, New Haven, CT 06510, USA; 3 Department of Anesthesia, Critical Care and Pain Medicine, Beth Israel Deaconess Medical Center, Boston, MA 02215, USA; 4 Yale Center for Analytical Sciences, Yale School of Public Health, New Haven, CT 06520, USA; 5 Department of Anesthesiology and Perioperative Medicine, Tufts Medical Center, Boston, MA 01803, USA

**Keywords:** pulmonary hypertension, postoperative pulmonary complications, postoperative mortality, perioperative outcomes, propensity score overlap weighting, Agency for Healthcare Research and Quality

## Abstract

**Background**: Pulmonary hypertension (PH) patients are at higher risk of postoperative complications. We analyzed the association of PH with 30-day postoperative pulmonary complications (PPCs). **Methods**: A single-center propensity score overlap weighting (OW) retrospective cohort study was conducted on 164 patients with a mean pulmonary artery pressure (mPAP) of >20 mmHg within 24 months of undergoing elective inpatient abdominal surgery or endoscopic procedures under general anesthesia and a control cohort (N = 1981). The primary outcome was PPCs, and the secondary outcomes were PPC sub-composites, namely respiratory failure (RF), pneumonia (PNA), aspiration pneumonia/pneumonitis (ASP), pulmonary embolism (PE), length of stay (LOS), and 30-day mortality. **Results**: PPCs were higher in the PH cohort (29.9% vs. 11.2%, *p* < 0.001). When sub-composites were analyzed, higher rates of RF (19.3% vs. 6.6%, *p* < 0.001) and PNA (11.2% vs. 5.7%, *p* = 0.01) were observed. After OW, PH was still associated with greater PPCs (RR 1.66, 95% CI (1.05–2.71), *p* = 0.036) and increased LOS (median 8.0 days vs. 4.9 days) but not 30-day mortality. Sub-cohort analysis showed no difference in PPCs between pre- and post-capillary PH patients. **Conclusions**: After covariate balancing, PH was associated with a higher risk for PPCs and prolonged LOS. This elevated PPC risk should be considered during preoperative risk assessment.

## 1. Introduction

Pulmonary hypertension (PH) is an adverse cardiopulmonary condition that is precipitated by a range of pathologic insults affecting the blood vessels of the lung [1]. Common symptoms of PH include exertional dyspnea and fatigue, induced by vascular alterations of the lungs (pre-capillary PH), and/or cardiac dysfunction (combined or post-capillary PH). The pathophysiological origin of this condition remains unclear, with thromboembolic phenomena, immunological compromise, connective tissue disorders, and parenchymal lung and heart disease constituting the spectrum of primary disorders eliciting PH [2]. Currently, a definitive diagnosis of PH requires right heart catheterization and a measured mean pulmonary arterial pressure (mPAP) of >20 mmHg with hemodynamic etiology (pre-capillary, post-capillary, or combined pre- and post-capillary) defined by comparative measurements of pulmonary arterial occlusion pressure (PAOP). Adverse perioperative outcomes have been observed in patients with PH, but it is less clear whether there is an association with increased 30-day postoperative pulmonary complications (PPCs) which occur with an incidence of 1 to 23% in surgical populations [3,4]. In 2005, Ramakrishna and colleagues observed that out of 144 PH patients who survived surgery, 60 (42%) had one or more perioperative morbid events, of which respiratory failure (28%), cardiac arrhythmia (12%), and congestive heart failure (11%) were the most common. Patients undergoing intermediate-to-high-risk surgery were observed to have a 2.5-fold increase in inpatient morbid events when compared to low-risk procedures. Increased post-anesthesia care unit respiratory depressive episodes and increased in-hospital PPCs in PH patients have been observed after noncardiac surgery [5]. Similarly, an increased risk for postoperative in-hospital pneumonia was observed in PH patients after cardiac valve surgery [6]. Given the increasing global trend toward shorter postoperative length of stay, it is important to characterize predictive factors for PPC in a 30-day timeframe, permitting more precise risk estimates for the purposes of preoperative risk stratification and postoperative surveillance [7]. Based on the elevated short-term perioperative risk observed in prior publications, we rationalized that a PH population would have persistently higher risk for 30-day PPCs. In this study, we sought to estimate and compare 30-day PPCs in a population of PH (mPAP > 20 mmHg) and controls undergoing abdominal surgical and endoscopic procedures. We selected abdominal procedures to avoid the higher 30-day PPCs incidence associated with thoracic and cardiac procedures. We hypothesized that, after covariate balancing, the diagnosis of PH would be associated with increased risk of 30-day PPCs, as demonstrated by the Agency for Healthcare Research and Quality (AHRQ) PPC composite outcome. For exploratory secondary outcome analyses, we investigated PH’s association with categorized sub-composites of infectious pneumonia (PNA), aspiration pneumonia/pneumonitis (ASP), respiratory failure (RF), pulmonary embolism (PE), and 30-day mortality. Differences in 30-day PPC outcomes in pre- and post-capillary PH variants have not been described. However, given the higher generalized morbidity and poorer survival observed in patients with pre-capillary PH, we rationalized that a higher 30-day PPC risk would be observed. Thus, we compared pre-capillary (pulmonary artery occlusion pressure (PAOP) < 15 mmHg) and post-capillary (PAOP > 15 mmHg) PH to explore differences between these distinct PH etiologies. 

## 2. Materials and Methods

This retrospective cohort analysis was approved by the Yale University Institutional Review Board (IRB#2000032516) and received a waiver of informed consent. We strove to adhere to the Strengthening the Reporting of Observational Studies in Epidemiology (STROBE) Statement on reporting observational studies. 

**Figure 1:**
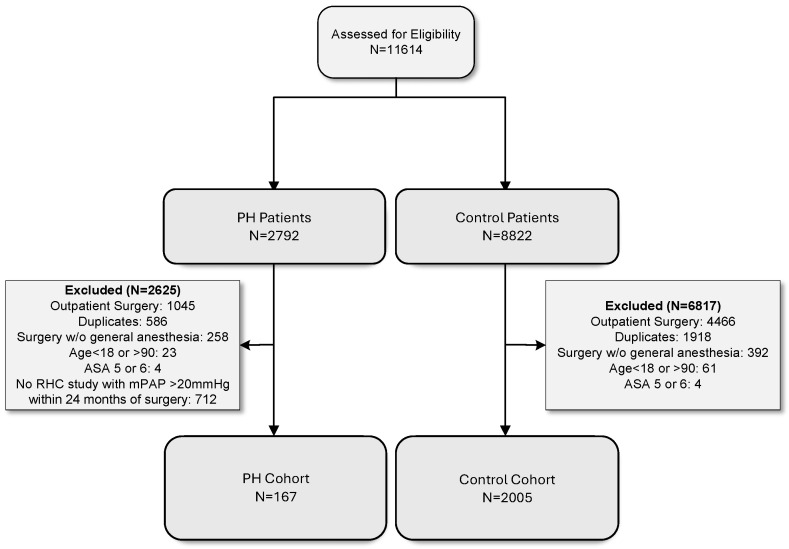
A flow diagram of patient selection criteria. Abbreviations: PH: pulmonary hypertension; ASA: American Society of Anesthesiologists Physical Status; RHC: right heart catheterization; mPAP: mean pulmonary artery pressure.

Study Population. Using a retrospective, single-center, comparative cohort design, we identified adult patients (18–90 years old) with International Classification of Disease version 10 (ICD-10) codes for PH (I27.0, I27.2) and American Society of Anesthesiologists Physical Statuses (ASA-PS) I to IV undergoing elective inpatient upper and lower abdominal surgery or gastrointestinal endoscopic procedures at a large quaternary care facility located in the United States between 1 January 2013 and 1 January 2020. The selection of these dates coincides with the introduction of a modern electronic record at the institution (Epic systems, Verona, WI, USA). Procedures were performed under general anesthesia with or without endotracheal intubation. After the initial identification of 2792 PH patients, we performed a manual electronic chart review and excluded patients without right heart catheterization data consistent with PH (mPAP > 20 mmHg) within 24 months of the procedure. A total of 167 patients were identified in the PH cohort. A control cohort of 2005 patients with similar ages, biological sex, and procedures were identified for comparison purposes (Figure 1). Due to incomplete outcomes, 27 patients (24 control; 3 PH) were removed from the final propensity score weighting analysis, and a total of 164 patients in the PH cohort and 1981 patients in the control cohort were identified.

Study Data Abstraction. Abstracted data were provided by the Joint Data Analytics Team (JDAT) at Yale School of Medicine/Yale New Haven Health. To ensure high-quality data, system data extraction processes required clear and consistent criteria for data inclusion and exclusion supported by the use of ICD-10 coding. We extracted demographical data, including age, biological sex, race, body mass index (BMI), Elixhauser comorbidity variables [8], and hospital site. We defined PH as a mean pulmonary arterial pressure (mPAP) > 20 mmHg on right heart catheterization (RHC) within two years of the surgical procedure. For the purposes of procedural risk matching, procedure severity was defined using the Surgical Outcome Risk Tool (SORT) v2 [9]. We used the number of Elixhauser comorbidity variables as a measure of frailty [10,11]. Moreover, 30-day PPC was defined by coding guidance provided by the AHRQ PPC composite outcomes (Table A1). We further divided the constituent codes into four separate sub-composites for detailed analysis of respiratory failure (RF), infectious pneumonia (PNA), aspiration pneumonia/pneumonitis (ASP), and pulmonary embolism (PE) to better understand their individual contribution to PPCs in the study population. The 30-day mortality, length of stay (LOS), and mortality-free discharge were also analyzed for both cohorts. Given the distinct differences between PH subtypes, we further sub-divided the PH cohort into pre-capillary PH (PAOP < 15 mmHg) and post-capillary PH (PAOP > 15 mmHg) to analyze the primary outcome.

Outcomes. Our primary outcome was PPCs, and secondary outcomes were RF, PNA, ASP, PE, LOS, and 30-day mortality. In a sub-cohort analysis, we compared pre-capillary PH and post-capillary PH to the primary and secondary outcomes.

Statistical Analysis. After verification of study variables, descriptive statistics were reported as mean and standard deviation (SD) or median and interquartile range (IQR) for continuous variables and frequency (%) for categorical variables. Initial assessment of the PH and control cohorts found significant systematic differences in baseline characteristics. We utilized the overlap weighting (OW) approach to optimize precise balance of the baseline covariates. OW is a propensity score (PS) method that attempts to mimic important attributes of randomized clinical trials. Briefly, OW assigns a weight to each patient that is proportional to the probability of that patient belonging to the opposite group. We chose the OW method because of the substantial baseline differences between the PH and control cohorts. OW has desirable statistical properties and leads to an exact balance on the mean of every measured covariate when the PS is estimated by a logistic regression [12]. The target population is the group of patients for whom the conclusions are drawn [13]; in our case, patients who undergo elective abdominal surgery or endoscopic procedures under general anesthesia comprise the target population. Due to incomplete outcomes, 27 patients (24 control; 3 PH) were removed before the final OW analysis. We fit a logistic regression model to estimate the PH propensity scores using baseline covariates—age, biological sex, race, BMI, number of Elixhauser comorbidities, procedural severity, and hospital location. The OW results were then generated and incorporated into all subsequent outcome analyses. Finally, we evaluated the group differences based on the standardized mean differences (SMD), where an SMD < 0.1 is usually deemed successful in achieving covariate balancing.

In the outcome analysis, for the binary outcomes PPC, RF, PNA, ASP, PE, and 30-day mortality, we used weighted log binomial regression to obtain the risk ratio (RR) for the RHC group compared to the controls. For the LOS outcome, we used weighted survival analysis, in which mortality-free discharge was the event and in-hospital patient deaths were censored. Weighted Cox proportional hazards model was used to obtain the hazard ratio (HR) of the mortality-free discharge for the PH cohort. Under this setting, a HR < 1 indicates a lower chance of mortality-free discharge. For the sub-cohort, no *p*-value adjustment was made due to the small sample size and lack of significant findings. We accepted a *p*-value of <0.05 to reject the null hypothesis. Data management and statistical analyses were carried out using R version 4.3.0.

## 3. Results

### 3.1. Baseline Characteristics of PH and Control Cohorts

The baseline characteristics are described in Table 1. The study included the PH cohort (N = 167) and the control cohort (N = 2005). The PH cohort was older (62 vs. 57 years old, *p* < 0.001), had more patients of a non-white ethnicity (44.5% vs. 26.8%, *p* < 0.001), a higher number of Elixhauser comorbidities (6.9 vs. 3.2, *p* < 0.001), and reduced procedural severity (minor or intermediate, 68.3% vs. 32.5%; major 15.6% vs. 43.0%; Xmajor/complex, 16.5% vs. 24.5%; *p* < 0.001). The types of surgeries in the PH vs. control cohorts were as follows: laparotomy (14% vs. 17%), laparoscopic surgery (21% vs. 54%), endoscopic procedures (65% vs. 26%), transvaginal surgeries (0.59% vs. 2.0%), and other surgeries (0.00% vs. 0.29%). For PH patients, more procedures were performed at the large quaternary care facility (72.6% vs. 61.9%, *p*= 0.02). Prior to propensity score overlap weighting adjustment, the PH cohort had higher 30-day PPC incidence (29.9% vs. 11.2%, *p* < 0.001). Regarding unadjusted secondary outcomes, the PH cohort was associated with significantly higher rates of RF (19.3% vs. 6.6%, *p* < 0.001) and PNA (11.2% vs. 5.7%, *p* = 0.010), but not ASP (3.7% vs. 1.7%, *p* = 0.118), PE (3.1% vs. 1.5%, *p* = 0.181), nor 30-day mortality (3.0% vs. 2.5%, *p* = 0.604). Among the baseline characteristics analyzed, male sex exhibited the strongest association with PPCs in patients with PH; however, the Pearson correlation coefficient was 0.068 (*p* = 0.38), signifying a very weak correlation. In terms of comorbidities, malignancy had the strongest association with PPCs, with a Pearson correlation coefficient of 0.20 (*p* = 0.008), which still represents a weak correlation.

### 3.2. Overlap-Weighted Outcome Analysis

Comparison between the PH cohort and the control cohort before and after OW adjustment is described in Table 2. Twenty-seven patients (24 control; 3 PH) were removed due to missing variables for OW analysis. Post-adjustment, the SMDs for each characteristic are all <0.01, indicating successful covariate balancing. Table 3 shows the findings after propensity-weighted matching. PH was associated with increased risk for 30-day PPC (risk ratio (RR) 1.66, 95% CI (1.05–2.71), *p* = 0.036). There were no differences in sub-composites RF (RR 1.68, 95% CI (0.90–3.28), *p* = 0.109), PNA (RR 1.21, 95% CI (0.57–2.65), *p* = 0.615), ASP (RR 1.63, 95% CI (0.38–8.28), *p* = 0.514), and PE (RR 1.20, 95% CI (0.29–5.28), *p* = 0.794). Survival analysis demonstrated that PH was associated with increased LOS (in days, median 8.0 vs. 4.9, 95% CI (6.8–9.1) vs. 4.9 (4.4–5.2)) and mortality-free discharge (HR 0.63, 95% CI (0.53–0.77), *p* = <0.001). These conclusions remain the same even after adjusting the *p*-values for multiple secondary outcomes.

### 3.3. Sub-Cohort Analysis: Comparisons of Pre-Capillary vs. Post-Capillary PH

The baseline characteristics of the sub-cohorts and OW model outcomes are described in Table 4. To analyze for between-group differences, the PH cohort was divided into pre-capillary PH (N = 47; mPAP > 20 mmHg and PAOP < 15 mmHg) and post-capillary PH (N = 116, mPAP > 20 mmHg and PAOP > 15 mmHg). We observed no difference in the primary outcome of PPCs (31.9% vs. 29.3%, *p* = 0.89) nor secondary outcomes of RF (21.3% vs. 18.6%, *p* = 0.86), PNA (4.3% vs. 14.2%, *p* = 0.126), ASP (6.4% vs. 2.7%, *p* = 0.36), and PE (2.1% vs. 3.5%, *p* = 1.0).

## 4. Discussion

**Figure 2:**
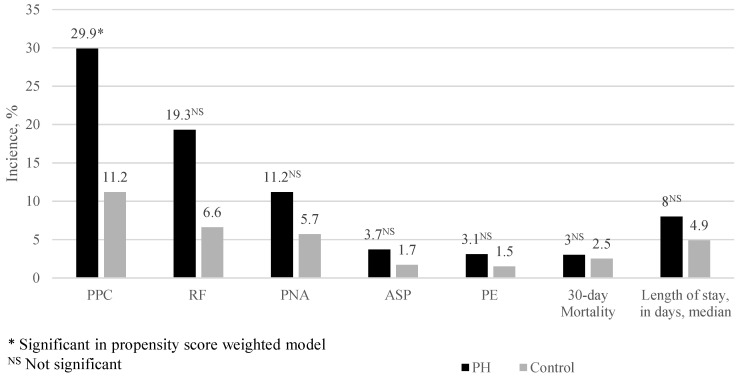
A comparison of PH vs. control cohorts’ unadjusted incidence of PPC and sub-composites.

In our study, we identified an unadjusted 29.9% risk for PPCs in the PH cohort vs. 11.2% in the control cohort after inpatient abdominal surgical and endoscopic procedures under general anesthesia. After OW covariate balancing, PH was associated with a 66% increase in the risk for PPCs in the 30 days following the procedure. Secondly, this increase in 30-day PPCs increased the LOS but not 30-day mortality. It has been estimated that 1% of the global population and up to 10% of those older than 65 years of age have PH, making it imperative to identify perioperative outcomes associated with this pathological condition [14]. Using a detailed composite measure of PPCs, secondary sub-composite analysis did not provide any guidance toward specific categories that most strongly contributed to the development of PPCs in PH patients. PH patients demonstrated higher incidences across all sub-composites when compared to controls (Figure 2). In addition to the short-term risks of perioperative administration of respiratory depressants and surgery-related compromise of respiratory mechanics, this lack of discrimination suggests two PH-related physiological consequences may contribute to a generalized 30-day PPC risk.

PH patients demonstrate an underlying dysregulated immune response [15]. Dysregulated inflammation has been associated with an increased propensity for perioperative complications, multiple organ dysfunction, and increased postoperative complications [16,17]. In a study of patients with pulmonary arterial hypertension and chronic thromboembolic-derived PH, circulating c-reactive protein (CRP), a marker for generalized inflammation, is significantly higher in PH patients compared to controls [18]. Furthermore, it is understood that underlying dysregulated immune responses may increase susceptibility to infectious pneumonia and favor the development of thromboembolic phenomenon [19]. This may be consistent with our observation of a 30-day postoperative pneumonia and pulmonary embolism prevalence nearly twice that of controls. Secondly, it may point to a general susceptibility to deleterious fluid shifts in the perioperative period. It is well understood that right ventricular dysfunction is common in PH patients during physiological stress [20,21]. Increasing serum natriuretic peptide, a validated measure of HF severity, has been associated with worse weaning outcomes in postoperative patients and is a measure of risk stratification in pulmonary arterial hypertension and a biomarker for PH-related right ventricular dysfunction [22,23,24,25]. Perioperative volume overload and positive cumulative fluid balance have been associated with increased morbidity and mortality risk in both general and surgical subpopulations [26].

Although limited by a small sample size, we did not observe a significant increase in 30-day mortality. The 30-day mortality after elective non-cardiac surgery in PH patients has been reported to be between 2% and 18% [2,27,28]. Both short- and long-term postoperative mortality have been observed to increase in patients who develop PPCs [29], and increased 90-day mortality risk has been demonstrated after abdominal surgery [30]. However, the LOS in the PH cohort was nearly 2-fold that of the controls, suggesting that although failure to rescue from perioperative complications is low, it does increase healthcare utilization. In the survival analysis, where mortality-free hospital discharge was compared, the PH cohort had a significantly lower likelihood of mortality-free discharge after OW adjustment. Thus, it is likely that PH increased the complexity and cost of required medical care in our study cohort. We did not identify a unique etiology contributing to the increased incidence of PPC in patients with PH, although postoperative respiratory failure, respiratory insufficiency and pneumonia comprised the majority of outcomes. This suggests that postoperative monitoring methods that enhance early recognition and treatment of these complications will likely improve patient safety in PH patients.

We did not observe a difference in PPCs between pre-capillary and post-capillary PH. This was a limited observation, given the small sample size of our study population, but surprising nonetheless, given the higher generalized morbidity and poorer survival exhibited by patients with pre-capillary PH [31,32].

Limitations. Our findings should be considered in the context of the potential confounding factors associated with retrospective cohort studies. To minimize convenience sampling, we performed random frequency matching stratified by age, biological sex, and procedure. We attempted to reduce misclassification bias by initial use of ICD-10 data followed by manual extraction of right heart catheterization data utilizing a cutoff value of mPAP > 20 mmHg for PH cohort inclusion [33]. The observed high incidence of PPCs in the control cohort may be attributable to (1) the exclusion of outpatient procedures and (2) the use of a broadly inclusive PPC composite measure. Furthermore, we used a 24-month window for RHC data, potentially reducing diagnostic precision for PH, although there is no available guidance regarding RHC surveillance criteria in PH populations. Due to incomplete data, we were unable to capture diagnostic group data for clinical classification of PH in our study population. Similarly, we were only able to utilize PAOP < or >15 mmHg to define pre- and post-capillary groups for exploratory sub-cohort analysis due to incomplete reporting of pulmonary vascular resistance on RHC reports. Although OW can achieve highly precise balancing and minimize between-group variance, it cannot adjust for unmeasured patient characteristics or unknown confounding variables at the time of the study.

## 5. Conclusions

This study reports that PH was associated with a 66% increased risk for 30-day PPCs when compared to controls undergoing abdominal surgical and endoscopic procedures under general anesthesia. This finding was associated with a significant increase in LOS but not 30-day mortality. Surprisingly, we did not observe differences between pre-capillary and post-capillary groups, suggesting that PH etiology may not be a strong contributing factor to 30-day PPCs. The significant increase in LOS after abdominal procedures strongly suggests that postoperative care utilization is increased in this population. The provided evidence offers perioperative physicians (1) improved insight when estimating postoperative risk in PH populations and (2) characterizes areas for improved postoperative surveillance and intervention. For example, RF and PNA constitute a significant portion of 30-day PPC risk in PH populations. This suggests that future studies should investigate (1) whether extended postoperative volume restriction strategies will reduce RF risk and (2) whether extended prophylactic perioperative antibiotic regimens or other nonpharmacological interventions can reduce PNA risk. In conclusion, these observations bring attention to the extended perioperative PPC risk profile in PH patients, the potential benefit of including PH in risk prediction tools and highlights the urgent need for ongoing identification of modifiable risk factors and evidence-based, protocolized postoperative care.

## Figures and Tables

**Table 1:** Comparisons of the baseline characteristics between control and PH cohorts.

	**Control** **(N = 2005)**	**PH Cohort** **(N = 167)**	***p*** -Value *	**Overall** **(N = 2172)**
Gender				
Female	1327 (66.2%)	104 (62.3%)	Chi-sq *p* = 0.348	1431 (65.9%)
Male	678 (33.8%)	63 (37.7%)		741 (34.1%)
Age in years				
Mean (SD)	57.4 (17.2)	62.2 (13.9)	*t*-test *p* < 0.001	57.8 (17.0)
Body Mass Index				
Mean (SD)	34.3 (112)	29.6 (8.87)	*t*-test *p* = 0.074	33.9 (108)
Missing	24 (1.2%)	3 (1.8%)		27 (1.2%)
Number of Elixhauser Comorbidities				
Mean (SD)	3.22 (2.56)	6.92 (1.98)	*t*-test *p* < 0.001	3.51 (2.70)
Ethnicity				
White	1467 (73.2%)	93 (55.7%)		1560 (71.8%)
Hispanic	166 (8.3%)	18 (10.8%)	Fisher *p* < 0.001	184 (8.5%)
Black	322 (16.1%)	47 (28.1%)		369 (17.0%)
Other	50 (2.5%)	9 (5.4%)		59 (2.7%)
ASA Class				
1–2	612 (37.5%)	3 (1.9%)	Chi-sq *p* < 0.001	615 (34.3%)
3–4	1019 (62.5%)	157 (98.1%)		1176 (65.7%)
Missing	374 (18.7%)	7 (4.2%)		381 (17.5%)
Procedural Severity **				
Minor	1 (0.0%)	1 (0.6%)		2 (0.1%)
Intermediate	652 (32.5%)	113 (67.7%)		765 (35.2%)
Major	867 (43.2%)	26 (15.6%)	Fisher *p* < 0.001	893 (41.1%)
Xmajor/Complex	485 (24.2%)	27 (16.2%)		512 (23.6%)
Hospital Location				
Community Hospital	757 (37.8%)	46 (27.5%)		803 (37.0%)
Endoscopy Center	2 (0.1%)	0 (0%)	Fisher *p* = 0.017	2 (0.1%)
Quaternary Care Facility	1246 (62.1%)	121 (72.5%)		1367 (62.9%)
Thirty-Day Mortality				
Yes	49 (2.4%)	5 (3.0%)		54 (2.5%)
No	1956 (97.6%)	162 (97.0%)	Fisher *p* = 0.604	2118 (97.5%)
Length of Stay (in days)				
Median [Q1, Q3]	3.2 [1.9, 5.9]	7.7 [4.2, 13.8]	Kruskal–Wallis test *p* < 0.001	3.23 [1.98, 6.43]

* *p*-value excludes missing values, ** determined by Surgical Outcome Risk Tool (SORT) v2 criteria. Abbreviations: PH, pulmonary hypertension; Q, quartile; SD, standard deviation.

**Table 2:** Comparison of PH versus control cohorts before and after propensity score overlap weighting adjustment.

	Before Overlap Weighting			After Overlap Weighting		
Variables	Control (N = 1981) *	PH (N = 164) *	SMD	Control (N = 1981) *	PH (N = 164) *	SMD
Age (years), mean (SD)	57.4 (17.18)	62.02 (13.72)	0.29	62.62 (15.65)	62.62 (14.10)	<0.01
Gender			0.08			<0.01
Female	1310 (66.1)	102.0 (62.2)		72.4 (60.8)	72.4 (60.8)	
Male	671.0 (33.9)	62.0 (37.8)		46.6 (39.2)	46.6 (39.2)	
Race			0.39			<0.01
White, non-Hispanic	1450 (73.2)	91.0 (55.5)		71.6 (60.1)	71.6 (60.1)	
Hispanic	165 (8.3)	18 (11.0)		12.0 (10.1)	12.0 (10.1)	
Black	317 (16.0)	46.0 (28.0)		30.1 (25.3)	30.1 (25.3)	
Other	49.0 (2.5)	9.0 (5.5)		5.3 (4.5)	5.3 (4.5)	
BMI, mean (SD)	34.2 (112.4)	29.6 (8.8)	0.06	29.6 (15.1)	29.6 (9.0)	<0.01
Number of Elixhauser comorbidities, mean (SD)	3.2 (2.55)	6.9 (1.97)	1.64	6.5 (2.77)	6.5 (1.83)	<0.01
Hospital Location			0.23			<0.01
Community Hospital	753 (38.0)	45 (27.4)		36.3 (30.5)	36.3 (30.5)	
Endoscopy Center	2.0 (0.1)	0.0 (0.0)		0.0 (0.0)	0.0 (0.0)	
Quaternary Care Facility	1226.0 (61.9)	119 (72.6)		82.7 (69.5)	82.7 (69.5)	
Procedure Severity			0.80			<0.01
Minor or Intermediate	644 (32.5)	112 (68.3)		76 (63.9)	76 (63.9)	
Major	852 (43.0)	25 (15.2)		21 (18.0)	21 (18.0)	
Xmajor/Complex	485 (24.5)	27 (16.5)		21 (18.2)	21 (18.2)	

Values are N (%) unless otherwise indicated. * 27 patients (24 in control cohort, 3 PH cohort) were removed due to missing variables for propensity score overlap weighting analysis. Abbreviations: PH, pulmonary hypertension; SMD; standardized mean difference; BMI, body mass index (kg/m^2^).

**Table 3:** Overlap-weighted propensity model findings of primary and secondary outcomes in the PH cohort.

Outcome	Unadjusted *			Propensity Overlap-Weighted Model **		
	PH	Control	*p*-Value	RR	95% CI	*p*-Value
PPC	49 (29.9%)	221 (11.2%)	<0.001	1.66	1.05–2.71	0.036
RF	31(19.3%)	131 (6.6%)	<0.001	1.68	0.90–3.28	0.109
PNA	18 (11.2%)	113 (5.7%)	0.010	1.21	0.57–2.65	0.615
ASP	6 (3.7%)	34 (1.7%)	0.118 (f)	1.63	0.38–8.28	0.514
PE	5 (3.1%)	30 (1.5%)	0.181 (f)	1.2	0.29–5.28	0.794
30-day mortality	5 (3.0%)	49 (2.5%)	0.604 (f)	1.23	0.43–2.76	0.651
Length of stay, median days (IQR)	8 (6.8, 9.1)	4.9 (4.4, 5.2)	<0.001	0.63	0.53–0.77	<0.001

* Chi-Square test; *p*-value excludes missing values, ** Propensity weighted by the following variables: age, biological sex, race, BMI, procedural severity, and number of Elixhauser covariates. (f) Fisher’s exact test applied. Abbreviations: PH, pulmonary hypertension; PPC, postoperative pulmonary complications; RF, respiratory failure; PNA, infectious pneumonia; ASP, aspiration pneumonia/pneumonitis; PE, pulmonary embolism; RR, relative risk; CI, confidence interval; IQR, interquartile range; BMI, body mass index.

**Table 4:** Baseline characteristics and postoperative outcomes of the PH cohort and comparison of outcome between pre- and post-capillary PH groups.

Variable	PH Group	Pre-Capillary	Post-Capillary	*p*-Value *
Age (years)	63.2 (13.9)	59.88 (14.8)	63.0 (13.5)	0.18
BMI (kg/m^2^)	29.6 (8.9)	27.8 (10.4)	30.2 (8.0)	0.12
Echocardiography				
Right Ventricular Systolic Pressure (mmHg)	52.9 (20.6)	54.3 (20.3)	52.2 (20.9)	0.59
Right Heart Catheterization				
Mean Right Atrial Pressure (mmHg)	13.2 (7.7)	9.5 (5.9)	14.6 (8.0)	<0.001
Right Ventricular Systolic Pressure (mmHg)	53.8 (18.9)	51.9 (19.8)	54.5 (18.6)	0.42
Pulmonary Artery Systolic Pressure (mmHg)	53.3 (18.4)	51.2 (19.9)	54.0 (17.7)	0.37
Pulmonary Artery Diastolic Pressure (mmHg)	25.0 (9.1)	21.5 (8.6)	26.4 (8.9)	0.002
Pulmonary Artery Mean Pressure (mmHg)	35.7 (11.6)	33.2 (11.4)	36.7 (11.4)	0.08
Pulmonary Artery Occlusion Pressure (mmHg)	19.1 (7.3)	11.35 (2.5)	22.2 (6.2)	<0.001
Pulmonary Artery Oxygen Saturation (%)	64.3 (9.6)	65.5 (8.5)	64.0 (9.8)	0.39
Fick Cardiac Output (L/min)	5.9 (2.5)	5.2 (1.9)	6.2 (2.6)	0.02
Calculated Cardiac Index	3.1 (1.2)	3.0 (1.2)	3.1 (1.1)	0.60
Primary and Secondary Outcomes **		Pre-capillary (N = 47)	Post-capillary (N = 116)	*p*-value
PPC		15 (31.9%)	34 (29.3%)	0.889
RF		10 (21.3%)	21 (18.6%)	0.863
PNA		2 (4.3%)	16 (14.2%)	0.126
ASP		3 (6.4%)	3 (2.7%)	0.36
PE		1 (2.1%)	4 (3.5%)	1

Figures in baseline characteristics sections are means followed by SD in parentheses unless otherwise indicated. * 2-side p-value after unadjusted t-test, equal variances assumed. ** 4 patients were excluded for missing pulmonary artery occlusion pressure. Abbreviations: PH, pulmonary hypertension; PPC, postoperative pulmonary complications; RF, respiratory failure; PNA, infectious pneumonia; ASP, aspiration pneumonia/pneumonitis; PE, pulmonary embolism, SD, standard deviation.

## Data Availability

We do not have publicly archived datasets analyzed or generated during the study.

## Appendix A

**Table A1:** Definition of postoperative pulmonary complications (PPC) and its sub-composites of respiratory failure (RF), infectious pneumonia (PNA), aspiration pneumonia/pneumonitis (ASP), and pulmonary embolism (PE) using International Classification of Disease version 10 (ICD-10) codes provided by the Agency for Healthcare Research and Quality (AHRQ).

AHRQ-PPC	ICD-10	Sub-Composite Inclusion
Transfusion-related acute lung injury	J95.84	AHRQ-RF
Respiratory complications not elsewhere classified	J95.85	AHRQ-RF
Ventilator associated pneumonia	J95.86	AHRQ-PNA
Post-procedural aspiration pneumonia	J95.87	AHRQ-ASP
Other complication of ventilator	J95.88	AHRQ-RF
Other intraoperative complications of respiratory system, not elsewhere classified	J95.89	AHRQ-RF
Other postprocedural complications and disorders of respiratory system, not elsewhere classified	J95.90	AHRQ-RF
Acute respiratory failure, unspecified whether with hypoxia or hypercapnia	J95.91	AHRQ-RF
Respiratory failure, unspecified, unspecified whether with hypoxia or hypercapnia	J95.92	AHRQ-RF
Respiratory failure, unspecified with hypoxia	J95.93	AHRQ-RF
Respiratory failure, unspecified with hypercapnia	J95.94	AHRQ-RF
Acute and chronic respiratory failure, unspecified whether with hypoxia or hypercapnia	J95.95	AHRQ-RF
Acute and chronic respiratory failure with hypoxia	J95.96	AHRQ-RF
Acute and chronic respiratory failure with hypercapnia	J95.97	AHRQ-RF
Respiratory disorders in diseases classified elsewhere	J95.98	AHRQ-RF
Pulmonary insufficiency following trauma and surgery	J95.99	AHRQ-RF
Acute postprocedural respiratory failure	J95.100	AHRQ-RF
Acute respiratory failure with hypoxia	J95.101	AHRQ-RF
Acute respiratory failure with hypercapnia	J95.102	AHRQ-RF
Acute pulmonary insufficiency following thoracic surgery	J95.103	AHRQ-RF
Acute pulmonary insufficiency following nonthoracic surgery	J95.104	AHRQ-RF
Chronic pulmonary insufficiency following surgery	J95.105	AHRQ-RF
Acute and chronic postprocedural respiratory failure	J95.106	AHRQ-RF
Acute respiratory distress syndrome	J95.107	AHRQ-RF
Chronic respiratory failure, unspecified whether with hypoxia or hypercapnia	J95.108	AHRQ-RF
Chronic respiratory failure with hypoxia	J95.109	AHRQ-RF
Chronic respiratory failure with hypercapnia	J95.110	AHRQ-RF
Respiratory arrest	J95.111	AHRQ-RF
Pneumonia due to streptococcus pneumonia	J95.112	AHRQ-PNA
Other bacterial pneumonia	J95.113	AHRQ-PNA
Pneumonia due to Pseudomonas	J95.114	AHRQ-PNA
Pneumonia due to streptococcus	J95.115	AHRQ-PNA
Pneumonia due to staphylococcus	J95.116	AHRQ-PNA
Pneumonia due to Methicillin susceptible Staphylococcus	J95.117	AHRQ-PNA
Pneumonia due to Methicillin susceptible Staphylococcus aureus	J95.118	AHRQ-PNA
Pneumonia due to Escherichia coli	J95.119	AHRQ-PNA
Pneumonia due to other Gram-negative bacteria	J95.120	AHRQ-PNA
Pneumonia due to other specified bacteria	J95.121	AHRQ-PNA
Unspecified bacterial pneumonia	J95.122	AHRQ-PNA
Pneumonia due to other specified infectious organisms	J95.123	AHRQ-PNA
Invasive pulmonary aspergillus	J95.124	AHRQ-PNA
Bronchopneumonia, unspecified organism	J95.125	AHRQ-PNA
Pneumonia, unspecified organism	J95.126	AHRQ-PNA
Pneumonia due to Klebsiella pneumonia	J95.127	AHRQ-PNA
Pneumonia due to other streptococci	J95.128	AHRQ-PNA
Pneumonitis due to solids and liquids	J95.129	AHRQ-ASP
Hypostatic pneumonia, unspecified organism	J95.130	AHRQ-PNA
Other ill-defined and unknown causes of morbidity and mortality	J95.131	
Respiratory conditions due to chemical fumes and vapors	J95.132	
Pneumonitis due to solids and liquids	J95.133	AHRQ-ASP
Pneumonitis due to inhalation of food and vomit	J95.134	AHRQ-ASP
Postprocedural pneumothorax	J95.135	
Septic pulmonary embolism without acute cor pulmonale	J95.136	AHRQ-PE
Other pulmonary embolism without acute cor pulmonale	J95.137	AHRQ-PE
Air embolism following infusion, transfusion and therapeutic injection, initial encounter	J95.138	AHRQ-PE
Complication of other artery following a procedure, not elsewhere classified, initial encounter	J95.139	
Complication of vein following a procedure, not elsewhere classified, initial encounter	J95.140	
Embolism due to cardiac prosthetic devices, implants and grafts, initial encounter	J95.141	AHRQ-PE
Embolism due to cardiac prosthetic devices, implants and grafts, initial encounter	J95.142	AHRQ-PE
Embolism due to vascular prosthetic devices, implants and grafts, initial encounter	J95.143	AHRQ-PE
Embolism due to vascular prosthetic devices, implants and grafts, initial encounter	J95.144	AHRQ-PE
